# Special Issue “Complex Dynamic System Modelling, Identification and Control”

**DOI:** 10.3390/e24030380

**Published:** 2022-03-08

**Authors:** Quanmin Zhu, Giuseppe Fusco, Jing Na, Weicun Zhang, Ahmad Taher Azar

**Affiliations:** 1 Department of Engineering Design and Mathematics, University of the West of England, Frenchy Campus Coldharbour Lane, Bristol BS16 1QY, UK; 2 Department of Electrical and Information Engineering, Universita degli Studi di Cassino e del Lazio Meridionale, 03043 Cassino, FR, Italy; fusco@unicas.it; 3 Faculty of Mechanical & Electrical Engineering, Kunming University of Science and Technology, No. 727 Jingming South Road, Chenggong, Kunming 650500, China; najing25@kust.edu.cn; 4 School of Automation and Electrical Engineering, University of Science and Technology Beijing, Beijing 100083, China; weicunzhang@ustb.edu.cn; 5 College of Computer & Information Sciences (CCIS), Prince Sultan University, Riyadh 11586, Saudi Arabia; aazar@psu.edu.sa or or; 6 Faculty of Computers and Artificial Intelligence, Benha University, Benha 13518, Egypt

## 1. Introduction

Systems are naturally or purposely formed with functional components and connection structures. The complicities could be induced from nonlinearity, dynamics, time delay, uncertainties, disturbances, irreversible processes, and those characteristics generally explained in other literature.

Modeling represents an innate intuition for humans to find rules or mechanisms that govern phenomena (a process/plant in a human-made system or a natural system, such as earth’s global climate, organisms, and the human brain). This is generally consistent with the journal titled Entropy, as the idea of entropy provides a mathematical way to encode/model the intuitive notion of which processes are obviously complex due to their irreversible characteristics, even though they would not violate the fundamental law of the conservation of energy. There are two predominant approaches to establish models, principle-based (e.g., information theory, statistic physics, statistical mechanics, etc.) analytical equations and data (measured and simulated) driven input/output fitted sets of regression numerical polynomials (most commonly called identification). Control is a way to improve a system behavior/performance by adding additional functional components and revising the system structure to form a closed-loop framework with adaptation and robustness to the uncertainties. Accordingly, modeling, identification, and control (MIC) is a cross-discipline from all engineering (human-made) systems to all natural scientific research discoveries.

This Special Issue (SI) encourages those emerging insights and approaches to provide concise/effective solutions in complex dynamic system modeling, identification, and control. The philosophy embedded in the SI is to seek simplicity (solutions) from complicity (problems). This Special Issue is a forum for presenting new and improved insight, methodologies, and techniques of MIC for complex systems that are challenging for research and (potentially) significant for a wide range of applications in real-world natural and engineering domains. Fundamentally, the papers should justify why the works have not been undertaken by the other colleagues and what the bottleneck issues have been for such research progression and applications.

## 2. The Expanded SI Publication List

This SI accepted/published 18 papers. Here, a brief summary is presented as an expanded content list for quick view of the SI.

Li et al. [1] propose a U-model-based two-degree-of-freedom internal model control (UTDF-IMC) structure with strength in nonlinear dynamic inversion and the separation of tracking design and robustness design. This approach can effectively accommodate modeling error and disturbance while removing those widely used linearization techniques for nonlinear plants/processes. To assure the expansion and applications, it analyses the key properties associated with the UTDF-IMC. For initial benchmark testing, computational experiments are conducted using MATLAB/Simulink for two mismatched linear and nonlinear plants. Further tests consider an industrial system, in which the IMC of a permanent magnet synchronous motor (PMSM) is simulated to demonstrate the effectiveness of the design procedure for potential industrial applications.

Kang et al. [2] consider the situations: Android devices are currently widely used in many fields, such as automatic control, embedded systems, the Internet of Things, and so on. At the same time, Android applications (apps) always use multiple permissions, and permissions can be abused by malicious apps that disclose users’ privacy or breach the secure storage of information. FlowDroid has been extensively studied as a novel and highly precise static taint analysis for Android applications. Aiming to resolve the problem of complex detection and false alarms in FlowDroid, an improved static detection method based on feature permission and risk rating is proposed. Firstly, the Chi-square test is used to extract correlated permissions related to malicious apps, and mutual information is used to cluster the permissions to generate feature permission clusters. Secondly, risk calculation method based on permissions and combinations of permissions are proposed to identify dangerous data flows. Experiments show that this method can significantly improve detection efficiency while maintaining the accuracy of dangerous data flow detection.

Jin et al. [3] consider trend prediction based on sensor data in a multi-sensor system as an important topic. As the number of sensors increases, we can measure and store more and more data. However, the increase in data has not effectively improved prediction performance. This paper focuses on this problem and presents a distributed predictor that can overcome unrelated data and sensor noise: First, the causality entropy is defined to calculate the measurement’s causality. Then, the series causality coefficient (SCC) is proposed to select the high causal measurement as the input data. To overcome the traditional deep learning network’s over-fitting to the sensor noise, the Bayesian method is used to obtain the weight distribution characteristics of the sub-predictor network. A multi-layer perceptron (MLP) is constructed as the fusion layer to fuse the results from different sub-predictors. The experiments were implemented to verify the effectiveness of the proposed method by meteorological data from Beijing. The results show that the proposed predictor can effectively model the multi-sensor system’s big measurement data to improve prediction performance.

Liang et al. [4] proposed a mechanistic kinetic model of cobalt–hydrogen electrochemical competition for the cobalt removal process in zinc hydrometallurgy. In addition, to overcome the parameter estimation difficulties arising from the model nonlinearities and the lack of information on the possible value ranges of parameters to be estimated, a constrained guided parameter estimation scheme was derived based on model equations and experimental data. The proposed model and the parameter estimation scheme have two advantages: (i) The model reflected for the first time the mechanism of the electrochemical competition between cobalt and hydrogen ions in the process of cobalt removal in zinc hydrometallurgy. (ii) The proposed constrained parameter estimation scheme did not depend on the information of the possible value ranges of parameters to be estimated. (iii) The constraint conditions provided in that scheme directly linked the experimental phenomenon metrics to the model parameters, thereby providing deeper insights into the model parameters for model users. Numerical experiments showed that the proposed constrained parameter estimation algorithm significantly improved the estimation efficiency. Meanwhile, the proposed cobalt–hydrogen electrochemical competition model allowed for accurate simulation of the impact of hydrogen ions on cobalt removal rate as well as simulation of the trend of hydrogen ion concentration, which would be helpful for the actual cobalt removal process in zinc hydrometallurgy.

Luan et al. [5] consider that an industrial process, namely the estimation of feeding composition, is important for analyzing production status and making control decisions. However, random errors or even gross ones inevitably contaminate the actual measurements. Feeding composition is conventionally obtained via discrete and low-rate artificial testing. To address these problems, a feeding composition estimation approach based on a data reconciliation procedure is developed. To improve the variable accuracy, a novel robust M-estimator is first proposed. Then, an iterative robust hierarchical data reconciliation and estimation strategy is applied to estimate the feeding composition. The feasibility and effectiveness of the estimation approach are verified on a fluidized bed roaster. The proposed M-estimator showed better overall performance.

Pan et al. [6] propose a data-driven fault diagnosis method using the deep convolutional neural network (DCNN). The DCNN is used to deal with sensor and actuator faults of robot joints, such as gain error, offset error, and malfunction for both sensors and actuators, and different fault types are diagnosed using the trained neural network. In order to achieve the above goal, the fused data of sensors and actuators are used, where both types of fault are described in one formulation. Then, the deep convolutional neural network is applied to learn characteristic features from the merged data to try to find discriminative information for each kind of fault. After that, the fully connected layer performs prediction work based on learned features. In order to verify the effectiveness of the proposed deep convolutional neural network model, different fault diagnosis methods, including support vector machine (SVM), artificial neural network (ANN), conventional neural network (CNN) using the LeNet-5 method, and long-term memory network (LTMN), are investigated and compared with the DCNN method. The results show that the DCNN fault diagnosis method can realize high fault recognition accuracy while needing less model training time.

Li et al. [7] investigates a critical hazard identification method for railway accident prevention. A new accident causation network is proposed to model the interaction between hazards and accidents. To realize consistency between the most likely and shortest causation paths in terms of hazards for accidents, a method for measuring the length between adjacent nodes is proposed, and the most likely causation path problem is first transformed to the shortest causation path problem. To identify critical hazard factors that should be alleviated for accident prevention, a novel critical hazard identification model is proposed based on a controllability analysis of hazards. Five critical hazard identification methods are proposed to select critical hazard nodes in an accident causality network. A comparison of results shows that the combination of an integer programming-based critical hazard identification method and the proposed weighted direction accident causality network considering length has the best performance in terms of accident prevention.

Han et al. [8] propose a new active fault tolerant control scheme based on active fault diagnosis to address the component/actuator faults for systems with state and input constraints. Firstly, the active fault diagnosis is composed of diagnostic observers, constant auxiliary signals, and separation hyperplanes, all of which are designed offline. In online applications, only a single diagnostic observer is activated to achieve fault detection and isolation. Compared with the traditional multi-observer parallel diagnosis methods, such a design is beneficial to improve the diagnostic efficiency. Secondly, the active fault tolerant control is composed of outer fault tolerant control, inner fault tolerant control, and a linear-programming-based interpolation control algorithm. The inner fault tolerant control is determined offline and satisfies the prescribed optimal control performance requirement. The outer fault tolerant control is used to enlarge the feasible region, and it needs to be determined online together with the interpolation optimization. In online applications, the updated state estimates trigger the adjustment of the interpolation algorithm, which in turn enables control reconfiguration by implicitly optimizing the dynamic convex combination of outer fault tolerant control and inner fault tolerant control. This control scheme contributes to further reducing the computational effort of traditional constrained predictive fault tolerant control methods. In addition, each pair of inner fault tolerant control and diagnostic observer is designed in an integrated manner to suppress the robust interaction of influences between estimation error and control error. The soft constraint method is further integrated to handle some cases that lead to constraint violations. The effectiveness of these designs is finally validated by a case study of a wastewater treatment plant model.

Zhang et al. [9] consider that spacecraft with large flexible appendages are characterized by multiple system modes. They suffer from inherent low-frequency disturbances in the operating environment that consequently result in considerable interference in the operational performance of the system. It is required that the control design ensures the system’s high pointing precision, and it is also necessary to suppress low-frequency resonant interference as well as take into account multiple performance criteria, such as attitude stability and bandwidth constraints. Aiming to address the comprehensive control problem of this kind of flexible spacecraft, a control strategy is proposed using a structured H-infinity controller with low complexity that was designed to meet multiple performance requirements, so as to reduce the project cost and implementation difficulty. According to the specific resonant mode of the system, the design strategy of adding an internal mode controller, a trap filter, and a series PID controller to the structured controller is proposed, so as to achieve the comprehensive control goals through cooperative control of multiple control modules. A spacecraft with flexible appendages (solar array) is presented as an illustrative example in which a weighted function was designed for each performance requirement of the system (namely robustness, stability, bandwidth limit, etc.), and a structured comprehensive performance matrix with multiple performance weights and decoupled outputs was constructed. A structured H-infinity controller meeting the comprehensive performance requirements is given, which provides a structured integrated control method with low complexity for large flexible systems that is convenient for engineering practice and provides a theoretical basis and reference examples for structured H-infinity control. The simulation results show that the proposed controller gives better control performance compared with the traditional H-infinity one and can successfully suppress the vibration of large flexible appendages at 0.12 Hz and 0.66 Hz.

Li et al. [10] examine the adaptive control of high-order nonlinear systems with strict-feedback form. An adaptive fixed-time control scheme is designed for nonlinear systems with unknown uncertainties. In the design process of a backstepping controller, the Lyapunov function, an effective controller, and adaptive law are constructed. Combined with the fixed-time Lyapunov stability criterion, it is proven that the proposed control scheme can ensure the stability of the error system in finite time, and the convergence time is independent of the initial condition. Finally, simulation results verify the effectiveness of the proposed control strategy.

Luo et al. [11] investigate the cluster-delay mean square consensus problem of a class of first-order nonlinear stochastic multi-agent systems with impulse time windows. Specifically, on the one hand, a discrete control mechanism (i.e., impulsive control) was applied in the system instead of a continuous one, which has the advantages of low control cost and high convergence speed. On the other hand, the existence of impulse time windows was considered when modeling the system, i.e., a single impulse appears randomly within a time window rather than an ideal fixed position. In addition, this paper also considers the influence of stochastic disturbances caused by fluctuations in the external environment. Then, based on algebraic graph theory and Lyapunov stability theory, some sufficiency conditions that the system must meet to reach the consensus state are given. Finally, we designed a simulation example to verify the feasibility of the obtained results.

Pang et al. [12] consider that the continuous development of spacecraft with large flexible structures has resulted in an increase in the mass and aspect ratio of launch vehicles, while the wide application of lightweight materials in the aerospace field has increased the flexible modes of launch vehicles. In order to solve the problem of deviation from the nominal control or even destabilization of the system caused by uncertainties, such as unknown or unmodelled dynamics, frequency perturbation of the flexible mode, changes in its own parameters, and external environmental disturbances during the flight of such large-scale flexible launch vehicles with simultaneous structural deformation, rigid-elastic coupling and multimodal vibrations, an improved adaptive augmentation control method based on model reference adaption, and spectral damping are proposed in this paper, including a basic PD controller, a reference model, and an adaptive gain adjustment based on spectral damping. The baseline PD controller was used for flight attitude control in the nominal state. In the non-nominal state, the spectral dampers in the adaptive gain adjustment law extracted and processed the high-frequency signal from the tracking error and control-command error between the reference model and the actual system to generate the adaptive gain. The adjustment gain was multiplied by the baseline controller gain to increase/decrease the overall gain of the system to improve the system’s performance and robust stability, so that the system had the ability to return to the nominal state when it was affected by various uncertainties and deviated from the nominal state or even destabilized.

Zhang et al. [13] address the problem of local fault (unknown input) reconstruction for interconnected systems. This contribution consists of a geometric method which solves the fault reconstruction (FR) problem based on observations and a differential algebraic concept. The fault diagnosis (FD) problem is tackled using the concept of the differential transcendence degree of a differential field extension and the algebraic observability. The goal is to examine whether the fault occurring in the low-level subsystem can be reconstructed correctly by the output at the high-level subsystem under given initial states. By introducing the fault as an additional state of the low subsystem, an observer based approached is proposed to estimate this new state. Particularly, the output of the lower subsystem is assumed unknown, and is considered as auxiliary output. Then, the auxiliary outputs are estimated by a sliding mode observer, which is generated by using global outputs and inverse techniques. After this, the estimated auxiliary outputs are employed as virtual sensors of the system to generate a reduced-order observer, which is capable of estimating the fault variable asymptotically. Thus, the purpose of multi-level fault reconstruction is achieved. Numerical simulations on an intensified heat exchanger are presented to illustrate the effectiveness of the proposed approach.

Azar et al. [14], an all-guest editor involved work, presents the robust stabilization and synchronization of a novel chaotic system. First, a novel chaotic system is presented, which is realized by implementing a sigmoidal function to generate the chaotic behavior. A bifurcation analysis is provided in which by varying three parameters of this chaotic system, the respective bifurcations plots are generated and evinced to analyze and verify when this system is in the stability region or in a chaotic regimen. Then, a robust controller is designed to drive the system variables from the chaotic regimen to stability so that these variables reach the equilibrium point in finite time. The robust controller is obtained by selecting an appropriate robust control Lyapunov function to obtain the resulting control law. For synchronization purposes, the novel chaotic system designed in this study is used as a drive and response system, considering that the error variable is implemented in a robust control Lyapunov function to drive this error variable to zero in finite time. In the control law design for stabilization and synchronization purposes, an extra state is provided to ensure that the saturated input sector condition must be mathematically tractable. A numerical experiment and simulation results are evinced, along with the respective discussion and conclusion.

Wang et al. [15] propose comprehensive fault diagnosis method of rolling bearing about noise interference, fault feature extraction, and identification. Based on complete ensemble empirical mode decomposition with adaptive noise (CEEMDAN), detrended fluctuation analysis (DFA), and improved wavelet thresholding, a denoising method of CEEMDAN-DFA-improved wavelet threshold function was presented to reduce the distortion of the noised signal. Based on quantum-behaved particle swarm optimization (QPSO), multiscale permutation entropy (MPE), and support vector machine (SVM), the QPSO-MPE-SVM method was presented to construct the fault-features sets and realize fault identification. Simulation and experimental platform verification showed that the proposed comprehensive diagnosis method can not only better remove the noise interference and maintain the original characteristics of the signal by CEEMDAN-DFA-improved wavelet threshold function, but also overcome overlapping MPE values by the QPSO-optimizing MPE parameters to separate the features of different fault types. The experimental results showed that the fault identification accuracy of the fault diagnosis can reach 95%, which is a great improvement compared with the existing methods.

Li et al. [16] propose a novel, adaptive, fixed-time neural network tracking control scheme for nonlinear interconnected systems. An adaptive backstepping technique is used to address unknown system uncertainties in the fixed-time settings. Neural networks are used to identify the unknown uncertainties. The study shows that, under the proposed control scheme, all states in the system can converge into small regions near zero with fixed-time convergence time via Lyapunov stability analysis. Finally, the simulation example is presented to demonstrate the effectiveness of the proposed approach. A step-by-step procedure for engineers in industry process applications is proposed.

Chen et al. [17] consider that the coupling between variables in the multi-input multi-output (MIMO) systems brings difficulties to the design of the controller. Aiming to address this problem, this paper combines the particle swarm optimization (PSO) with the coefficient diagram method (CDM) and proposes a robust controller design strategy for the MIMO systems. The decoupling problem is transformed into a compensator parameter optimization problem, and PSO optimizes the compensator parameters to reduce the coupling effect in the MIMO systems. For the MIMO system with measurement noise, the effectiveness of CDM in processing measurement noise is analyzed. This paper gives the control design steps of the MIMO systems. Finally, simulation experiments of four typical MIMO systems demonstrate the effectiveness of the proposed method.

Liu et al. [18] consider that pulsars, especially X-ray pulsars detectable for small-size detectors, are highly accurate natural clocks, suggesting potential applications, such as interplanetary navigation control. Due to various complex cosmic background noise, the original pulsar signals, namely photon sequences, observed by detectors have low signal-to-noise ratios (SNRs) that obstruct practical use. This paper presents a pulsar denoising strategy developed based on the variational mode decomposition (VMD) approach. This is in fact the initial work of the authors’ interplanetary navigation control research. The original pulsar signals are decomposed into intrinsic mode functions (IMFs) via VMD, by which the Gaussian noise contaminating the pulsar signals can be attenuated because of the filtering effect during signal decomposition and reconstruction. Comparison experiments based on both simulation and HEASARC-archived X-ray pulsar signals are carried out to validate the effectiveness of the proposed pulsar denoising strategy.

## 3. Conclusions

In conclusion, the SI has witnessed and promoted great interest in <Complex Dynamic System Modelling, Identification and Control>. As always, the research topics associated with this SI are quite widely demanded, from academic research to real applications, particularly in those manmade systems (e.g., engineering products). Therefore, the guest editors hope that the readers can benefit from these published articles, the meaningful concepts and insights presented, emerging techniques, and inspiration for their future research and publications.

Once again, the guest editors wish to show their heartfelt gratitude to all the support and passion devoted to the SI from the authors, the publisher, and many others.
